# Holocene ecosystem and temperature development inferred from invertebrate remains in Zminje Jezero (Dinaric Alps, Montenegro)

**DOI:** 10.1007/s10933-024-00334-y

**Published:** 2024-08-06

**Authors:** Noé R. M. M. Schmidhauser, Walter Finsinger, Eleonora Cagliero, Oliver Heiri

**Affiliations:** 1 Geoecology, Department of Environmental Sciences, University of Basel, 4056 Basel, Switzerland; 2 Institute of Geography, University of Bern, Hallerstrasse 12, 3012 Bern, Switzerland; 3 Oeschger Centre for Climate Change Research, University of Bern, 3012 Bern, Switzerland; 4 ISEM, CNRS, IRD, University of Montpellier, Montpellier, France; 5 Department of Land, Environment, Agriculture and Forestry (TeSAF), University of Padova, Viale dell’Università, 16, 35020 Legnaro, PD, Italy

**Keywords:** Lake, Balkans, Sediment, Chironomids, Temperature reconstruction

## Abstract

Lake Zminje Jezero (1535 m a.s.l.) in Montenegro was studied for chironomid and other aquatic invertebrate remains in a sediment sequence dating back to 12,000 calibrated ^14^C years before present (cal yr BP), providing, to our knowledge, the first lake-sediment record studied for chironomids and other associated chitinous aquatic invertebrate remains in the Dinaric Alps. Changes in chironomid and other invertebrate remains along the record make it possible to constrain changes of relevant environmental variables for aquatic invertebrates in the lake including temperature, oxygen availability, trophic status and water depth. The results suggest moderate changes in trophic conditions with chironomid assemblages indicating meso- to eutrophic conditions during the analysed interval. Invertebrate assemblages were typical for lakes with relatively high oxygen availability with a minor trend during the middle to late Holocene to conditions observed in lakes that are more hypoxic. A change in temperature is suggested in the earlier part of the record when the share of chironomid taxa adapted to warmer conditions increased, at the Younger Dryas to Holocene transition, whereas later no particularly pronounced shifts in temperature-sensitive taxa were observed. July air temperatures were estimated based on chironomid assemblages using a transfer function based on calibration data consisting of 117 lakes sampled in the Swiss Alps and northern Switzerland. The results suggest that temperatures rapidly increased by 5 °C at the onset of the Holocene (11,500 cal yr BP) leading to a relatively warm early to mid-Holocene and thereafter slightly decreased during the late Holocene. Reconstructed temperatures are discussed together with a previously published pollen record for our study site and are broadly consistent with other chironomid-based temperature reconstructions from Central, Eastern and Southern Europe. From ca. 3000 cal yr BP sedimentation rates increased and from ca. 500 cal yr BP onwards pollen data suggest that the vegetation and lake catchment were affected by human activities, possibly influencing chironomid and other invertebrate assemblages in the lake and thereby also reconstructed temperatures. Overall, our results show that combining analyses of chironomid and other invertebrate assemblages can provide valuable insights into long-term environmental changes and can provide temperature reconstructions for small mountain lakes in the Balkans, such as Zminje Jezero.

**Supplementary Information:**

The online version contains supplementary material available at 10.1007/s10933-024-00334-y.

## Introduction

The Balkan Peninsula is characterized by diverse habitat types and a long history of human presence. However, in comparison to other European regions, only a limited number of palaeoecological and particularly lake-sediment studies are available from some parts of the Balkans such as the Dinaric Alps (Finsinger et al. 2017). Lake-sediments are excellent natural archives for reconstructing past environmental conditions in and around lakes (Gregory-Eaves and Smol 2024). Remains of a wide range of organisms can be preserved in lake-sediments such as chironomid head capsules, diatom valves, cladoceran remains, mandibles from different macroinvertebrates or pollen, other plant remains and fungal spores (Walker 1987; Ursenbacher et al. 2020; Courtney-Mustaphi et al. 2024). These remains provide an avenue for reconstructing changes in lacustrine community and ecosystem composition and, indirectly, for reconstructing and constraining changes in environmental conditions in lakes and their catchments.

Chironomids (non-biting midges) are a family of true flies (Diptera). After hatching from the egg, chironomids live as larvae in the uppermost sediments or on hard substrates in freshwater ecosystems or in other habitats such as water-logged soils, peats and dung (Walker 1987). When chironomid larvae die or moult, the head capsules are well preserved in the sediments (Walker 2001). Chironomids are abundant, present nearly everywhere (ubiquitous), persistent and have, as family, a wide range of tolerance to environmental change (Brooks 2003; Campbell et al. 2017). These traits, combined with the narrow environmental range that some species have (Lencioni and Rossaro 2005), makes them ideal indicators to reconstruct past environmental conditions. Because of rapid generation times and the ability of the adults to fly, changes in the assemblage as a response to environmental change can be considered instantaneous on multidecadal and longer time scales (Brooks et al. 2007). In small lakes, the distribution of chironomid taxa has been shown to be related to temperature, nutrients, pH, oxygen, salinity and other variables (Walker 1987; Brooks et al. 2007), with temperature often seen as a primary variable driving chironomid assemblages on longer time scales (Heiri and Lotter 2005). Changes in trophic status, often resulting from land-use activities, can also strongly influence the composition of chironomid assemblages causing, for example, increased nutrient availability and reduced oxygen concentrations (Heiri et al. 2003). In this context, chitinous remains of other aquatic invertebrate groups can provide valuable supporting information for interpreting chironomid records (Courtney-Mustaphi et al. 2024). For example, Ursenbacher et al. (2020) have shown that the abundance of chironomid remains relative to other invertebrates such as Chaoboridae, Trichoptera, Ephemeroptera, and oribatid mites can provide crucial information on changes in deep-water oxygen conditions in lakes. These remains are easily sorted from samples prepared for chironomid analysis during processing and mounting and can therefore be counted during analysis of chironomid samples.

From Europe, a large number of chironomid records are now available that cover the Lateglacial (Heiri and Millet 2005; Samartin et al. 2017) and the Holocene period (Ilyashuk et al. 2011; Tóth et al. 2015). These records have provided detailed insights on past ecosystem dynamics and chironomid response to climatic changes and early human impacts (Taylor et al. 2013; Perret-Gentil et al. 2024). Furthermore, several of these records have provided the basis for estimating changes in summer temperatures based on the close relationship between summer temperature and chironomid distribution (Heiri et al. 2011). So far, most of the chironomid-based temperature reconstructions in Europe have shown an increase in temperature of 3–5 °C from the Younger Dryas (YD) to Holocene transition (centred around 11,500 cal yr BP), as well as a Holocene thermal maximum reconstructed between ca. 10,000 and 5000 cal yr BP. This is often followed by a slow decrease in temperature from the mid- to late Holocene (Ilyashuk et al. 2011; Heiri et al. 2015; Tóth et al. 2015; Samartin et al. 2017). However, from Southern European mountain ranges only few such records are available (Tóth et al. 2015; Samartin et al. 2017; Jimenez-Moreno et al. 2023) and to our knowledge none from the Balkans.

Here we provide the first record of chironomid and other macroinvertebrate subfossils from a small mountain lake (Zminje Jezero) in the Dinaric Alps, a mountain range separating the continental Balkan Peninsula from the Adriatic Sea. The aims of this study were to:

1. reconstruct changes in the chironomid and other macroinvertebrate assemblages over the past 12,300 years in and around Zminje Jezero;
2. assess possible variations of in-lake factors (changes in trophic state, deep-water oxygen availability, and water depth) based on the chironomid and other invertebrate assemblages;
3. explore the new chironomid record to assess its potential to develop a first Holocene temperature reconstruction for the western Balkan region; and
4. compare the developed temperature reconstruction with other paleoenvironmental and palaeotemperature evidences from southern Central, Southern, and Southeastern Europe.

### Study site

**Fig. 1:**
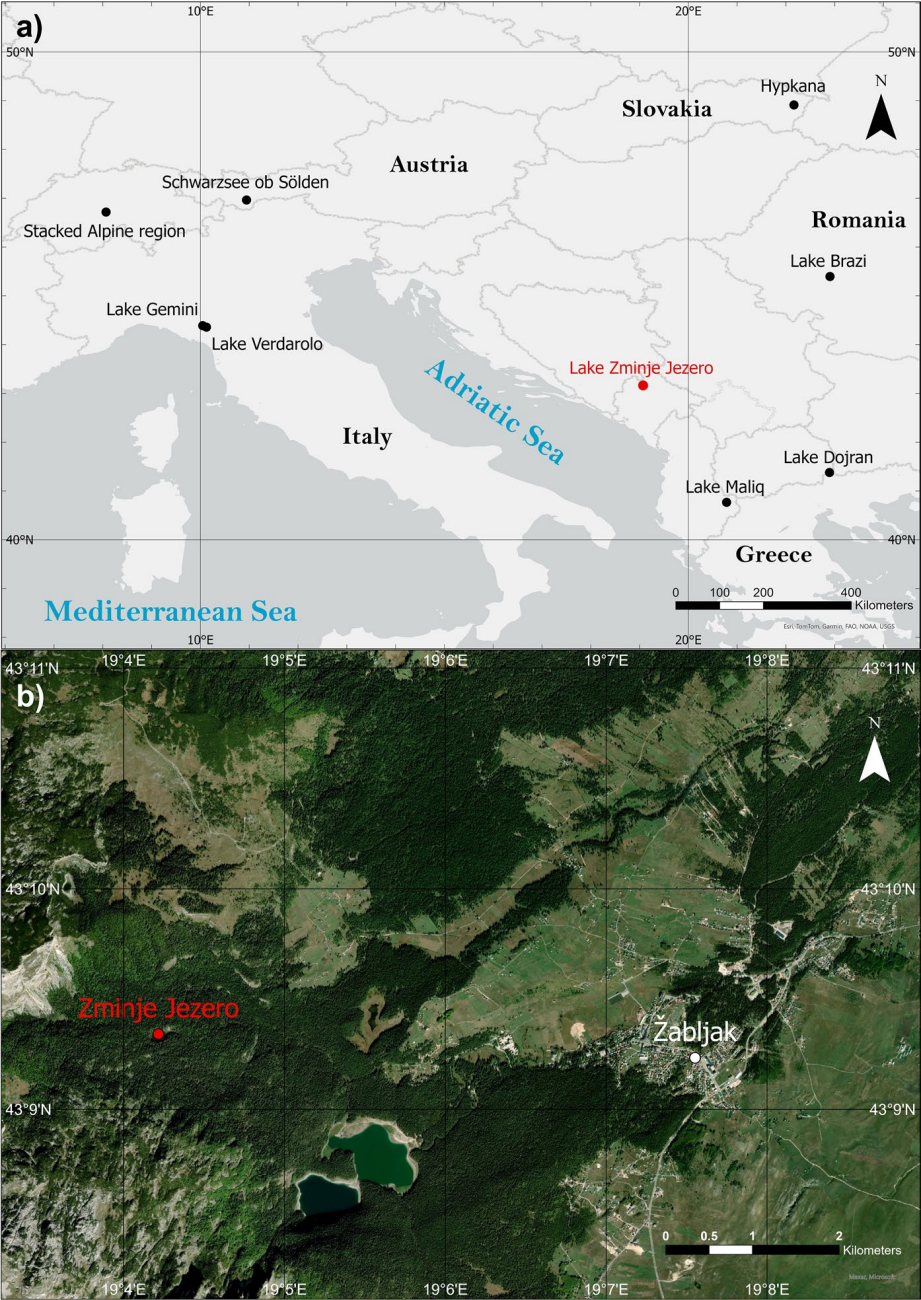
**a** Location of Zminje Jezero and the different study sites with chironomid-, pollen- and MBT-CBT-based temperature reconstructions mentioned in the discussion. The study sites are: Hypkana (Hájková et al. 2016), Lake Brazi (Tóth et al. 2015), Lake Gemini and Verdarolo (Samartin et al. 2017), Schwarzsee ob Sölden (Ilyashuk et al. 2011), a stacking of different lakes in the Alpine region (Heiri et al. 2015), Lake Maliq (Bordon et al. 2009) and Lake Dojran (Thienemann et al. 2017). Map data from Esri, HERE, Garmin, FAO, NOAA, USGS, © OpenStreetMap contributors, and the GIS User Community and available under https://www.arcgis.com/home/item.html?id=91b43cb1be4b418eafd522348a618531. The map was created using ArcGIS® software by Esri. **b** Location of Zminje Jezero (red dot) in the Dinaric Alps, Montenegro. Map data from Esri, Maxar, Earthstar Geographics, and the GIS User Community and available under https://www.arcgis.com/home/item.html?id=10df2279f9684e4a9f6a7f08febac2a9. The map was created using ArcGIS® software by Esri

Zminje Jezero is a lake of glacial origin situated in the central part of the Dinaric Alps (Durmitor Massif, Montenegro; Fig. 1), a mountain range that is made almost entirely from Mesozoic sedimentary rocks (Gachev and Mitkov 2019). The lake is located on limestone bedrock at 1535 m a.s.l. (43°09′21″ N, 19°04′14″ E). A small stream drains the water to a wider lake (Crno Jezero, 1460 m a.s.l.). Dense mixed forests dominated by *Picea abies* and *Abies alba* with lesser amounts of *Fagus sylvatica* and *Acer* sp. presently surround the lake (Cagliero et al. 2023). The lake is situated ca. 400 m below treeline (around 1950 m a.s.l.), which is formed by *Pinus mugo*, *Juniperus communis*, and *Picea abies* (Cagliero et al. 2023). Today, the lake is nearly 190 m long and 100 m wide with a surface area of around 1.2 ha and a maximum depth of 9.5 m. pH at the surface was 9.5, surface water conductivity 293 µS cm^−1^ and the lake had a Secchi depth of 1.5 m during fieldwork (September 2019). The lake shore is characterized by wetland vegetation, mainly *Molinia caerulea* and *Comarum palustre*. Climate is continental with a mean annual air temperature of 4.6 °C, mean July air temperature of 13.9 °C (Burić et al. 2014) and mean annual precipitation of ca. 1450 mm at Žabljak (1450 m a.s.l.; Djurović 2012), which is situated ca. 4 km from Zminje Jezero.

## Materials and methods

### Sampling

Surface sediments were sampled with an UWITEC gravity corer (6 cm diameter) in September 2017 and in September 2019. In September 2019, two parallel cores were taken with a Livingstone piston corer with 6 cm diameter and 1-m long barrels. The coring site is situated nearly in the middle and deepest part of the lake (9.2–9.3 m water depth). Core segments were split longitudinally in the laboratory and correlated to a continuous sequence based on lithological marker layers and XRF analyses as described in Cagliero et al. (2023). Several visually identified turbidite layers were considered to be instantaneous sedimentation events that do not represent long-term sedimentation and were omitted from the continuous composite sequence.

### Dating

The chronology of the sediment record (Fig. S1, Supplementary material 1) is constrained by 19 ^14^C dates from terrestrial plant macrofossils (Cagliero et al. 2023), the sediment surface (2019), the onset of the Holocene as apparent in the pollen assemblages (dated to 11,500 ± 250 cal yr BP; Giesecke et al. 2014) and the *Ambrosia* pollen increase (1950 ± 30 CE) (Table S1, Supplementary material 1). The first pollen appearance of the neophyte *Ambrosia* was dated around 1950 CE in the Southern Alps (Tinner et al. 1998; van der Knaap et al. 2000). This broadly coincides with results from the Dinaric Alps, where the mean sample ages of *Ambrosia*’s first pollen appearance have been dated to ca. 1930 ± 10 CE (Cagliero et al. 2022) and ca. 1940 ± 20 CE (Cagliero et al. 2023), thereby granting its use as a reliable, albeit biostratigraphic, age-control point. Radiocarbon (^14^C) samples were analyzed at Isotoptech Zrt. in Debrecen (Hungary) and at the Poznan Radiocarbon Laboratory (Poland) using Accelerator Mass Spectrometry (AMS). ^14^C ages were calibrated to calendar years BP using the IntCal20 dataset (Reimer et al. 2020) prior to modelling the age-depth relationship with RBacon v2.4.1 (Blaauw and Christen 2011) following Cagliero et al. (2023). Sample ages of this chronology differ slightly (< 60 cal yr BP) from the one by Cagliero et al. (2023), which included for the topmost 17.5 cm a chronology derived from short-lived radionuclides (^210^Pb and ^137^Cs).

### Chironomid, aquatic macroinvertebrate and macrophyte analyses

In total, 79 samples (2–8 cm^3^ wet sediment) were taken every 8 cm for the analysis of chironomid and other invertebrate remains. Samples were washed through a 100 µm mesh sieve. Macroinvertebrate fossils (including chironomid head capsules) and selected macrophyte remains were then picked with fine forceps from the sieve residue in a Bogorov counting tray under a stereomicroscope (Leica, 30–50× magnification). Picked remains were transferred to a microscope slide and mounted in Euparal mounting medium.

Macroinvertebrate fossils were identified under a compound microscope (Leica, 100–400× magnification). Chironomids were identified to genus level or, whenever possible, to species morphotype level, while other macroinvertebrates and a few selected aquatic macrophyte remains were identified to a coarser taxonomic level. Half chironomid head capsules were counted as half individuals to provide a conservative estimation. For the other macroinvertebrate remains, individual body parts (frons, frontoclypeus, labrum, pair of mandibles) were used to estimate the minimum number of individuals. Literature used for identifying chironomids included Brooks et al. (2007), Schmid (1993), Andersen et al. (2013) and Rieradevall and Brooks (2001). To identify other invertebrate remains, we used the following literature: Vandekerkhove et al. (2004) for cladoceran ephippia, Francis (2001) for bryozoan statoblasts, Solhøy (2001) for oribatid mites, Gelorini et al. (2011) for Rhabdocoela and Courtney-Mustaphi et al. (2024) as well as the slide collection of mounted invertebrate specimens of the Geoecology group (University of Basel) for Ephemeroptera, Plecoptera, *Sialis* (Sialidae), Trichoptera, Ceratopogonidae, Amphipoda, Coleoptera, Lepidoptera, Odonata, Sciaridae and Ostracoda. Charophyte oogonia were identified following Haas (1994), fern sporangia following Piasecki (2022) and testate amoebae following Mitchell et al. (2008). Chironomids were grouped into categories (terrestrial and stream, littoral, profundal) according to their preferred habitats following Brooks et al. (2007), Andersen et al. (2013), Janecek et al. (2017), Vallenduuk and Moller Pillot (2007), and Moller Pillot (2009, 2013). Invertebrate remains were classified into planktonic/benthic groups according to their preferred habitats following Thorp and Rogers (2014). Abundances of the main chitinous invertebrate remains are presented as percentages. Abundances of Rhabdocoela cocoons, testate amoeba, charophyte oogonia, *Procladius* pupal thoracic horns and fern sporangia are presented as counts per sample.

### Numerical analyses

Sedimentation rate was first calculated at 0.5 cm resolution using the age-depth model. To smooth the estimates of sedimentation rate, we applied a running average filter at 8 cm resolution. Influx of chironomid head capsules was calculated by multiplying the smoothed sedimentation rates (cm year^−1^) with chironomid concentrations (head capsules cm^−3^), as the smoothed sedimentation rates were considered more realistic than unsmoothed values.

For some samples, mostly between 22.5 and 323.5 cm, the recommended number of 40–45 chironomid head capsules (Heiri and Lotter 2001) was not reached. Thus, for temperature reconstruction and other numerical analyses, adjacent samples were combined to reach a minimum of 40 head capsules per sample. Zonation was based on stratigraphically constrained hierarchical cluster analysis (CONISS; Grimm 1987) using square-root transformed chironomid percentages and Euclidean distance. The number of significant zones was determined using the broken-stick model (Bennett 1996). To summarize overall variations in chironomid assemblages, a Detrended Correspondence Analysis (DCA) was conducted from square-root transformed chironomid percentages. To assess changes in deep-water oxygen conditions of Zminje Jezero, we conducted a Canonical Correspondence Analysis (CCA) based on a modern invertebrate dataset from 36 Swiss lakes (Ursenbacher et al. 2020). This dataset consisted of count data of aquatic invertebrate taxa such as aquatic insect remains, cladoceran ephippia, bryozoan statoblasts and oribatid mites (Acari) as well as associated environmental data. Sample scores of the first CCA axis of this analysis are closely correlated to deep-water oxygen concentrations. We passively added the Zminje Jezero invertebrate samples to the CCA of Ursenbacher et al. (2020) and considered variations of the passively added samples towards higher CCA axis 1 scores to represent changes in invertebrate composition towards oxygen-richer conditions. CONISS was calculated using the rioja v0.9–15.1 package (Juggins 2015) and ordinations using the vegan v2.6-2 package (Oksanen et al. 2020) in the R environment (R Core Team 2021).

July air temperature estimates were developed from the chironomid record using a temperature-inference model (transfer function) based on the Swiss chironomid-temperature calibration dataset (Heiri and Lotter 2010). The dataset consists of modern (surface sample) chironomid data and associated mean July air temperature estimates from 117 lakes of which 15 were deleted as outliers as described in Heiri and Lotter (2010). The dataset covers a mean July air temperature range of 5–18.4 °C and was selected since it comes from a Central European mountain range (the Alps) that is biogeographically similar to the Dinaric Alps and includes many sites that lie on carbonate bedrock, as Zminje Jezero. Also, it includes the majority of chironomid taxa expected in late Pleistocene and Holocene lake-sediments from Western, Eastern, and Central Europe as well as in Southern European mountain lakes (Heiri and Lotter 2010). The calibration dataset was used at the taxonomic resolution described in Heiri et al. (2011) although some additional taxonomic amalgamation was necessary to achieve the same taxonomic resolution in the Zminje Jezero and training-set data. Categories merged included *Micropsectra contracta*-type and *Micropsectra insignilobus*-type; *Psectrocladius sordidellus*-type, *Psectrocladius barbatipes*-type, *Psectrocladius bisectus*-type and *Psectrocladius psilopterus-*type; *Cricotopus albiforceps*-type and *Cricotopus* indet.; and *Chironomus plumosus-*type and *Einfeldia pagana*-type. *Paratanytarsus* individuals without mandibles in the Zminje Jezero data were merged with *Paratanytarsus penicillatus*-type, since this was clearly the most abundant *Paratanytarsus* morphotype in the record. The reconstruction was made using weighted averaging partial least squares (WAPLS) regression (ter Braak and Juggins 1993) and square-root transformed percentages using the program C2 (Juggins 2007). The model with two WAPLS components was selected because it had a smaller root mean square error of prediction and higher r^2^ than the model with one WAPLS component, if assessed using cross validation with 9999 bootstrapping cycles. Data for the Swiss calibration dataset were taken from Heiri et al. (2019).

## Results

### Chironomids, macroinvertebrate assemblages and macrophytes

A total of 3485 chironomid head capsules were isolated from the sediments that encompass the past 12,300 years. A small fraction (n = 88; c. 2.5% of the total) could not be identified to a detailed taxonomic level due to poor preservation. The 79 analyzed samples were combined to 41 samples for ecological and numerical analyses to ensure a minimum of 40 identified head capsules per sample.

**Fig. 2:**
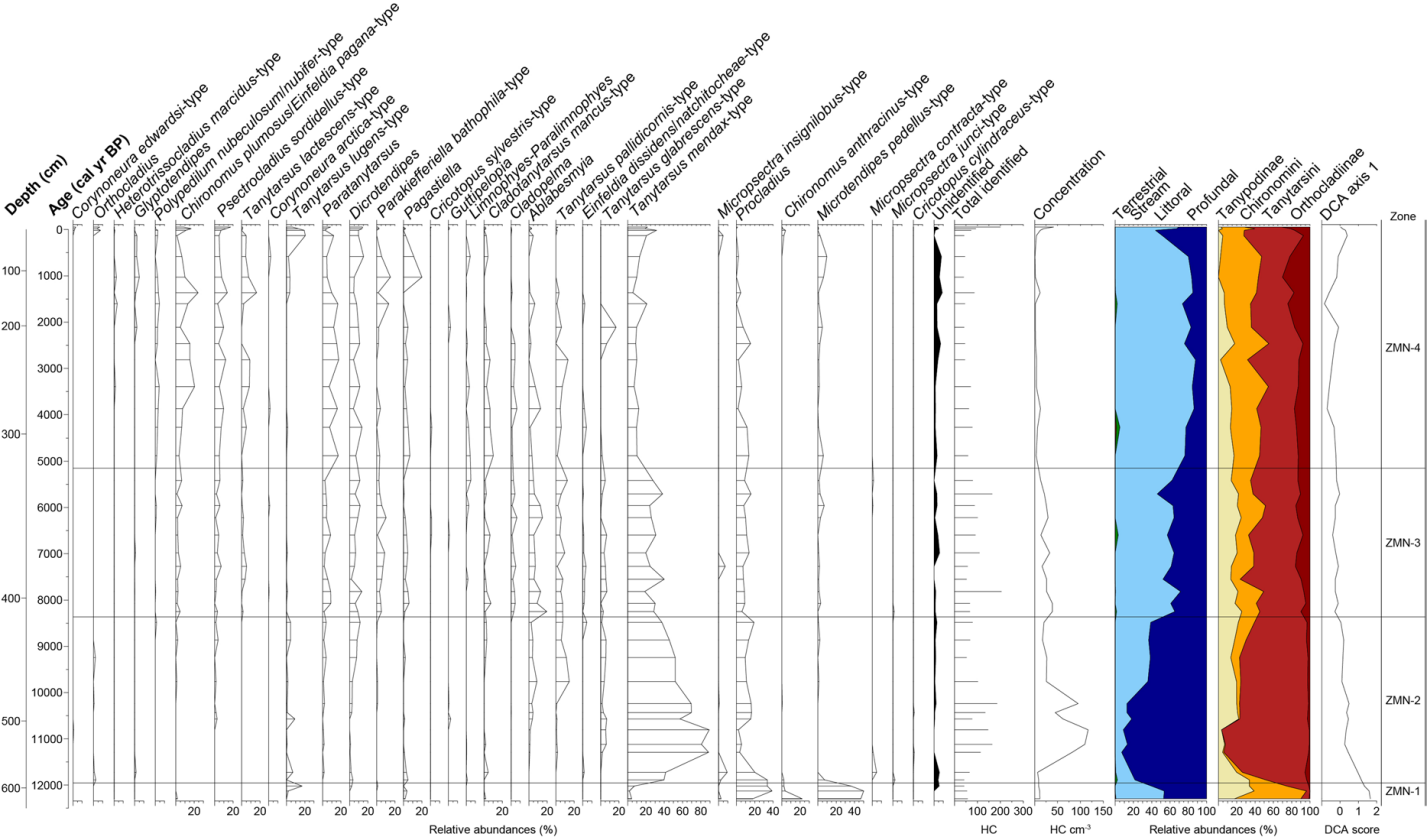
Simplified chironomid percentage diagram of Zminje Jezero, showing identified chironomid taxa that occurred in at least four samples. To the right, the abundances of chironomids by tribe or subfamily and by preferred habitat type (terrestrial and stream, littoral, profundal) are shown, along with DCA axis 1 scores of the chironomid assemblages. The horizontal lines represent the statistically significant CONISS zone boundaries for the chironomid-assemblage zones (ZMN-1 to ZMN-4)

Head capsules belonging to 66 different chironomid taxa were identified. The most frequent taxon was *Tanytarsus mendax*-type (Fig. 2). CONISS zonation identified four statistically significant zones (ZMN-1 to ZMN-4). Chironomid concentrations were highest (up to 100 head capsules cm^−3^) around 11,000–10,200 cal yr BP (zone ZMN-2, Fig. 2). Concentrations were also high in the youngest samples (up to 40 capsules cm^−3^) but did not exceed earlier values. From bottom to top, a reduction of the subfamily Tanypodinae and an increase of the subfamily Orthocladiinae is apparent. Also, an overall increase in taxa that prefer littoral habitats can be observed from 11,500 to 500 cal yr BP.

**Fig. 3:**
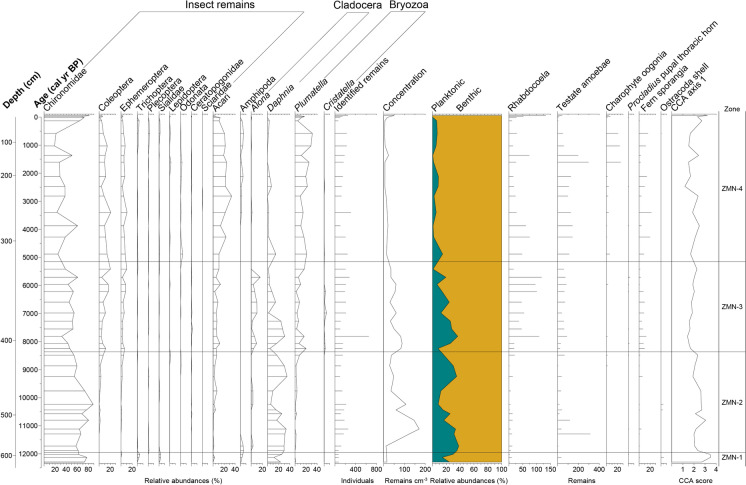
Simplified percentage diagram of invertebrate remains from Zminje Jezero (showing only taxa that occurred in at least three samples). Abundances of Rhabdocoela, testate amoebae, charophyte oogonia, *Procladius* pupal thoracic horns, fern sporangia and Ostracoda shells are shown as remains per sample. A classification of taxa according to the preferred habitat type (planktonic/benthic) and the first axis score of the CCA following Ursenbacher et al. (2020), representing changes in invertebrate assemblages indicative of variations in deepwater oxygen concentrations, are also shown. The horizontal lines represent the statistically significant CONISS zone boundaries for the chironomid-assemblage zones (ZMN-1 to ZMN-4)

A total of 11,987 invertebrate individuals or macrophyte fragments (including chironomid head capsules) were identified and amalgamated to the same 41 samples as the chironomid head capsules. Fossil remains belonging to 26 different invertebrate groups were identified. The most predominant were Chironomidae followed by oribatid mites with *Plumatella* also present at high abundances (Fig. 3). Being mostly driven by the concentration of Chironomidae, changes in overall abundance of invertebrate remains resembles the ones of chironomids, with higher concentrations in zones ZMN-2 and ZMN-3. Also, the relative importance of planktonic groups seems to decline along the record, with higher values in ZMN-1 to ZMN-3 and lowest values in ZMN-4. For easier comparison, changes in the invertebrate assemblages and macrophytes are described below, in the same zones (ZMN-1 to ZMN-4) as the chironomid-assemblage description.

### ZMN-1 (12,300–11,960 cal yr BP; 613.5–593 cm)

*Procladius*, *Chironomus anthracinus*-type and *Microtendipes pedellus*-type are present at high abundances. The proportions of these taxa decline when approaching the zone boundary to ZMN-2. *Pagastiella*, *Tanytarsus mendax*-type and *Tanytarsus lugens*-type are also present at lower abundances.

Macroinvertebrate assemblages are dominated by Chironomidae and *Daphnia*, with noticeable abundances of Trichoptera, Sialidae, and Amphipoda.

### ZMN-2 (11,960–8370 cal yr BP; 593–416.5 cm)

*Tanytarsus mendax*-type and *Procladius* are present at high abundances throughout the zone. A range of taxa such as *Dicrotendipes*, *Einfeldia dissidens*/*natchitocheae*-type, *Cladotanytarsus mancus*-type, *Ablabesmyia*, *Tanytarsus pallidicornis*-type, *Tanytarsus glabrescens*-type, *Paratanytarsus*, *Chironomus plumosus*/*Einfeldia pagana*-type, and *Psectrocladius sordidellus*-type appear in this zone and are present at noticeable abundances. *Tanytarsus lugens*-type observed in zone ZMN-1 is still present but at lower abundances.

Abundances of Chironomidae and *Daphnia* ephippia remain high and those of Trichoptera, Sialidae, and Amphipoda decline. Some taxonomic groups slightly increase such as Coleoptera, Ephemeroptera, Acari and *Plumatella*. Testate amoebae show a major peak between 11,300 and 10,800 cal yr BP.

### ZMN-3 (8370–5150 cal yr BP; 416.5–323.5 cm)

*Tanytarsus mendax*-type and *Procladius* are still co-dominant and the abundance of *Microtendipes pedellus*-type increases slightly. Taxa which appeared in ZMN-2 remain present throughout this zone in noticeable abundances although *Tanytarsus lugens*-type only occurs occasionally. New taxa such as *Cladopelma*, *Tanytarsus lactescens*-type, *Limnophyes-Paralimnophyes* increase in abundance for the first time in this zone.

The proportion of Coleoptera, Ephemeroptera, Acari and *Plumatella* increases in this zone. Among the Cladocera, *Alona* reaches highest abundance whereas *Daphnia* decreases slowly, while the abundance of Rhabdocoela, testate amoebae and fern sporangia visibly increases.

### ZMN-4 (5150 cal yr BP-today; 323.5–0 cm)

More taxa become co-dominant next to *Procladius* and *Tanytarsus mendax*-type. This is the case for *Paratanytarsus*, *Dicrotendipes*, *Tanytarsus lactescens*-type, *Psectrocladius sordidellus*-type, and *Chironomus plumosus*/*Einfeldia pagana*-type*,* which are all present at relatively high abundances. New taxa increase such as *Polypedilum nubeculosum*/*nubifer*-type, *Glyptotendipes*, and *Heterotrissocladius marcidus*-type. From 1000 cal yr BP onwards, additional new taxa increase in abundance, such as *Corynoneura edwardsi*-type and *Orthocladius* together with taxa that were already present in zones ZMN-1 or ZMN-2, such as *Tanytarsus lugens*-type, *Chironomus anthracinus*-type or *Microtendipes pedellus*-type.

*Acari* and *Plumatella* are present at their maximum abundances in this zone. Coleoptera and Ephemeroptera have similar abundances as in ZMN-3. Amphipoda appear again at similar abundances as in ZMN-1 and *Daphnia* shows the lowest abundance, whereas Rhabdocoela, testate amoebae and fern sporangia proportions remain high. Charophyte oogonia show an increase in the second half of this zone.

### DCA, CCA, sedimentation rate and chironomid influx

DCA axis 1 scores based on chironomid assemblages show highest values in the lowest samples (Fig. 2). The scores decrease rapidly in the early Holocene, and then stay at relatively low values throughout the early Holocene and zone ZMN-2. A minor decreasing trend to even lower values is apparent for most of the mid- to late Holocene (ZMN-3 to ZMN-4) but scores increase again in the uppermost ca. 1000 years (ZMN-4) to similar values as in the early Holocene, suggesting a reversal in the trajectory of chironomid assemblages. Overall, the DCA indicates that the most distinct assemblage changes occurred at the YD to Holocene transition and in the earliest Holocene.

In the CCA of the modern invertebrate dataset of Ursenbacher et al. (2020), axis 1 correlates strongly with deep-water oxygen availability in modern Swiss lakes. The CCA axis 1 scores of the passively added samples are highest (up to 3.5) before 12,000 cal yr BP indicating the occurrence of highest deep-water oxygen availability during the YD. Overall, assemblages from Zminje Jezero seem to remain indicative of high O_2_ concentrations throughout the entire sequence, since their CCA axis 1 scores (> 1.2; Figs. 3, 5) are in the upper half of the range of CCA axis 1 scores (− 1.2 to 3.1) from extant Swiss lakes examined by Ursenbacher et al. (2020). Nonetheless, we can observe a trend towards lower values during the mid- to late Holocene (Zones ZMN-3 to ZMN-4).

**Fig. 4:**
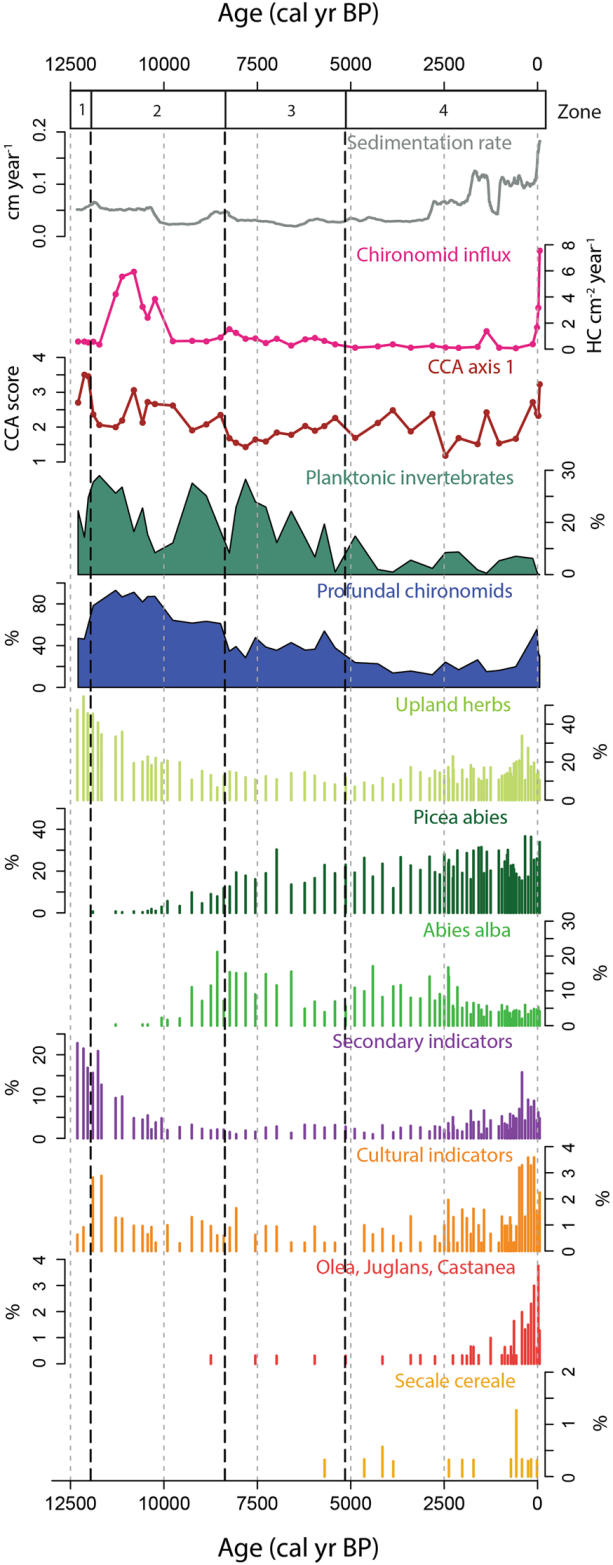
Records representing local environmental and ecosystem change at Zminje Jezero, including sedimentation rates, chironomid influx, axis 1 scores of a CCA of modern invertebrate data (Ursenbacher et al. 2020) with Zminje Jezero data added passively, percentages of planktonic invertebrates and profundal chironomids, and pollen percentages of selected pollen types and pollen groups (from Cagliero et al. 2023). Secondary indicators in the pollen data indicate the sum of adventives and apophytes and cultural indicators, the sum of Cerealia-type and *Plantago lanceolata-*type pollen (see Cagliero et al. 2023 for details). *Olea*, *Juglans* and *Castanea* represent woody crops that were cultivated at lower altitudes along the coast of the Adriatic Sea. The vertical black dashed lines indicate the statistically significant CONISS zone boundaries of the chironomid-assemblage zones (ZMN-1 to ZMN-4)

Sedimentation rates in Zminje Jezero (Fig. 4) remained moderately high from 12,300 to 10,400 cal yr BP (higher than 0.05 cm year^−1^). Between 10,000 and 3000 cal yr BP, sedimentation rates were low and stable (below 0.05 cm year^−1^). From 3000 cal yr BP onward sedimentation rates rise progressively to reach a maximum of 0.18 cm year^−1^ at the sediment surface.

Chironomid influx values (Fig. 4) are highest between 11,500 and 10,000 cal yr BP (with a local maximum of 6 HC cm^−2^ year^−1^) and in the topmost section of the core (8 HC cm^−2^ year^−1^in the topmost sample). Otherwise, values are low with a minimum between 1000 and 500 cal yr BP (down to 0.07 HC cm^−2^ year^−1^). Local maxima are apparent around 8250 cal yr BP and 1350 cal yr BP when influx values are slightly higher (up to 1.53 HC cm^−2^ year^−1^).

### Chironomid inferred July air temperature reconstruction

**Fig. 5:**
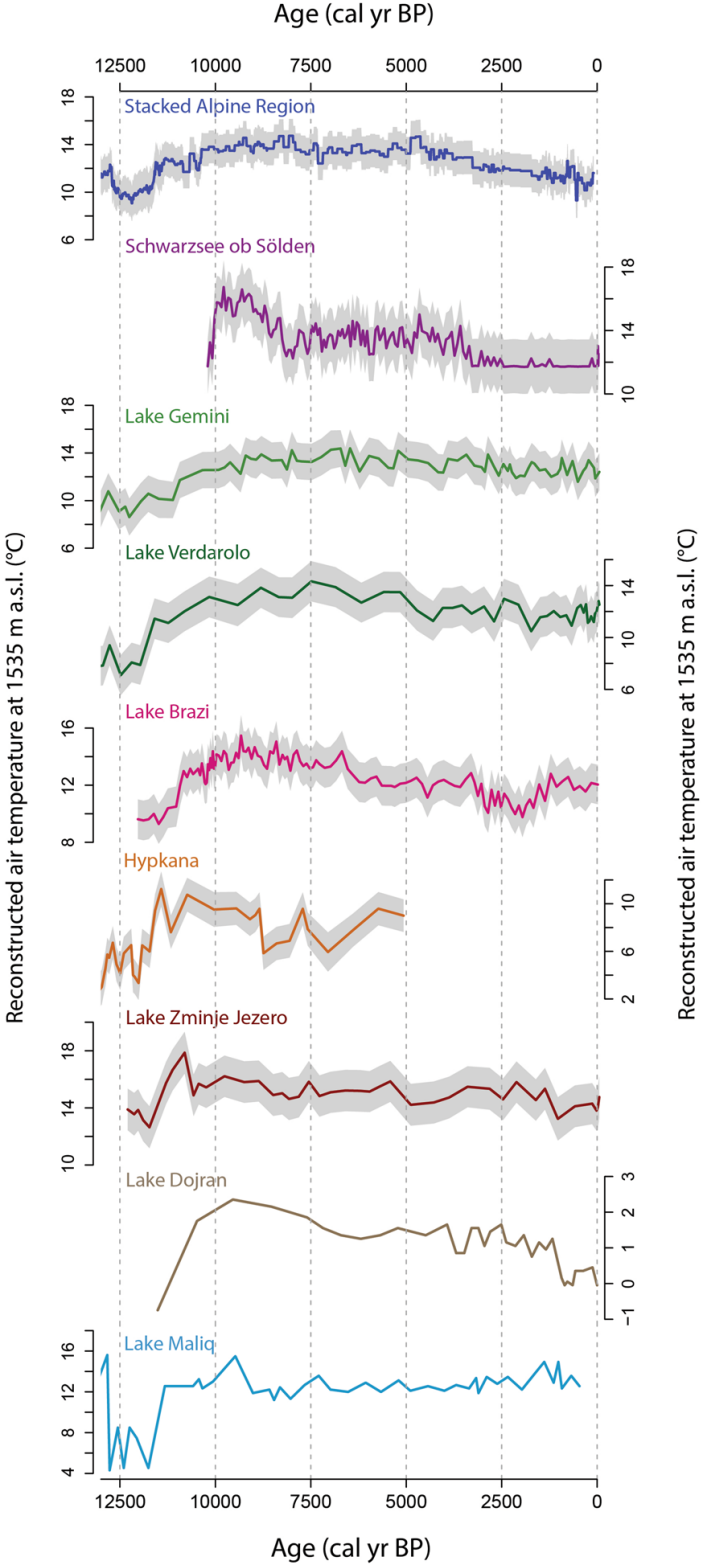
Comparison of the Zminje Jezero chironomid-based July air temperature reconstruction with other chironomid-based July air temperature reconstructions, the pollen-based temperature reconstruction of Lake Maliq (mean temperature of the warmest month) and the MBT-CBT-based temperature reconstruction of Lake Dojran (annual MAT). Uncertainties (eSEPs) in the chironomid-based reconstructions are indicated by the shaded area. All temperature reconstructions were corrected to the altitude of Zminje Jezero (1535 m a.s.l.) using an altitudinal lapse rate of 0.6 °C 100 m^−1^. The location of the different sites is shown in Fig. 1

Reconstructed mean July air temperatures (Fig. 5) are lowest between 12,300 and 11,700 cal yr BP with values below 14 °C. Temperature then rises to reach a short-lived maximum near 18 °C around 10,800 cal yr BP. Afterwards, temperatures rapidly decrease and then remain relatively stable until 2000 cal yr BP with values between 14.6 and 16.2 °C except for a centennial-scale period with slightly lower temperatures down to 14.2 °C from 5000 to 4000 cal yr BP. From 2000 years to 1000 cal yr BP a progressive decrease in temperature is observed, with the lowest values of 13.2 °C at 1000 cal yr BP. Over the past 1000 years, temperatures show a slight increase to reach 14.8 °C in the uppermost sample.

## Discussion

### Chironomid and macroinvertebrate assemblages and lake development

#### The end of the Younger *Dryas*

Low concentrations of chironomid and other aquatic invertebrate remains characterize ZMN-1 (12,300–11,960 cal yr BP), possibly a consequence of low in-lake productivity affecting macroinvertebrate assemblages. High proportions of *Procladius* are observed, a generalist taxon that occurs under varying climatic and environmental conditions (Brooks et al. 2007; Heiri et al. 2011). A similar ecology characterizes *Tanytarsus mendax*-type, a morphotype which was found throughout the core and is characteristic for intermediate cool to warm lakes (Saether 1979; Brooks et al. 2007; Heiri et al. 2011). In ZMN-1 *Tanytarsus mendax*-type is found at lower abundances than in other zones, possibly due to cooler conditions that favor other chironomid taxa.

The temperature in and around the lake was cool to intermediate during the YD. This is shown by a chironomid assemblage co-dominated by *Chironomus anthracinus*-type typical of cool to intermediate temperatures in small lakes (Heiri et al. 2011) and by *Microtendipes pedellus*-type that can also be found in relatively cool conditions (Bolland et al. 2021). This is also in accordance with the presence in smaller numbers of *Tanytarsus lugens*-type, also primarily living under cool conditions (Heiri et al. 2011). *Tanytarsus mendax*-type and *Procladius* are able to survive over a broad range of trophic conditions (Lotter et al. 1997, 1998). However, other taxa having narrower ecological requirements show that the lake was probably oligotrophic to mesotrophic during the YD. For example, *Chironomus anthracinus*-type is found in oligo- to mesotrophic conditions (Saether 1979; Brooks et al. 2007) and *Microtendipes pedellus*-type mainly in oligo- to mesotrophic conditions (Brooks et al. 2007; Moller Pillot 2009) but can also be found in eutrophic lakes (Lotter et al. 1998). Also, *Tanytarsus lugens*-type, present at lower abundance, is typical for oligotrophic conditions (Brooks et al. 2007). Of the macroinvertebrate remains found, the presence of Sialidae could indicate lower lake levels or a pronounced contribution of remains from near-shore areas, as these aquatic insects are usually considered indicative of littoral conditions and muddy lake bottoms (Lemdahl 2000). However, relatively high abundances of chironomids that can survive in profundal conditions and of planktonic invertebrate remains (Fig. 4) suggest that the lake was not particularly shallow during this period.

#### The Younger *Dryas*/Holocene transition and the early Holocene

In zone ZMN-2 (11,960–8370 cal yr BP) several chironomids often found in relatively cool conditions in small Central and Southern European mountain lakes (*Chironomus anthracinus*-type, *Microtendipes pedellus*-type; Heiri and Lotter 2010; Samartin et al. 2017) diminish in abundance or disappear whereas taxa with a wide temperature distribution remain at high abundances (*Procladius*) or increase (*Tanytarsus mendax*-type). We observe higher concentrations of chironomids which suggests overall improved conditions for this family or higher habitat diversity. High chironomid influx values from 2 to 6 HC cm^−2^ year^−1^ can be seen in this zone between 11,500 and 10,000 cal yr BP (Fig. 4). This may be the result of an increase of the chironomid abundance during this interval due to changing environmental conditions. Many taxa appear that were previously absent or only present at low abundances. These include *Dicrotendipes*, *Einfeldia dissidens*/*natchitocheae*-type, *Cladotanytarsus mancus*-type, *Ablabesmyia*, *Tanytarsus pallidicornis*-type, *Tanytarsus glabrescens*-type, *Paratanytarsus*, *Chironomus plumosus*/*Einfeldia pagana*-type and *Psectrocladius sordidellus-*type. *Cladotanytarsus mancus*-type, *Ablabesmyia, Tanytarsus glabrescens*-type and *Tanytarsus pallidicornis*-type have a relatively wide distribution with respect to temperature but tend to be more abundant in warmer lakes (Heiri et al. 2011) and this would agree with some warming compared to the zone below. However, *Tanytarsus lugens*-type, often an indicator of cold conditions, remains at low abundances in some samples and *Psectrocladius sordidellus*-type, which has a very wide distribution but is also able to live in colder conditions (Heiri et al. 2011), increases in the upper part of this zone. This may indicate some minor variation of temperature. Most of the taxa listed above, which are present in this zone at high abundances, are indicators of mesotrophic or eutrophic conditions with the exception of some species within *Paratanytarsus* and *Tanytarsus lugens*-type which are indicators of more oligotrophic conditions (Brooks et al. 2007). This suggests more nutrient-rich conditions of the lake compared to the previous zone.

Higher concentrations of aquatic invertebrate remains (Fig. 3) suggest improved conditions for most of these groups, although no abrupt change can be seen at the transition from ZMN-1 to ZMN-2. Instead, a slow increase in some macroinvertebrates, or the disappearance of groups present at low proportions in zone ZMN-1, such as Trichoptera, Sialidae, and Amphipoda, is observed. Chironomids that can colonize the profundal of lakes are present at relatively high abundances, and planktonic invertebrate remains are common (Fig. 4), suggesting a lake of at least intermediate depth.

#### The middle Holocene

At the transition to ZMN-3 (8370–5150 cal yr BP), taxa which appeared in ZMN-2 increase slowly or remain at similar abundances. Only *Tanytarsus lugens*-type, which previously persisted at low abundances disappears from the record. A few new taxa like *Cladopelma*, *Tanytarsus lactescens*-type and *Limnophyes-Paralimnophyes* increase or occur for the first time. *Cladopelma* and *Limnophyes-Paralimnophyes* are typical for intermediate to warm July air temperatures in Central European mountain lakes (Heiri et al. 2011). *Tanytarsus lactescens*-type, which first appears in this zone, indicates temperate climatic conditions (Heiri and Lotter 2010). The appearance of chironomid taxa typical for mesotrophic (*Cladopelma*) and eutrophic conditions (*Tanytarsus lactescens*-type) according to Brooks et al. (2007), show that the mesotrophic to eutrophic conditions found in zone ZMN-2 persisted in zone ZMN-3. However, the general decrease of taxa that can survive in profundal environments in favour of taxa restricted to the littoral of lakes (Fig. 2) could indicate a tendency to lower oxygen availability in the deepest part of the lake (Walker et al. 1993). Only chironomid taxa adapted to low oxygen availability, for example by optimizing or regulating the oxygen uptake, can survive in deeper water with seasonally reduced oxygen concentration. The genera *Chironomus*, *Ablabesmyia* and *Procladius* have these abilities (Brodersen et al. 2004) and this possibly explains why these taxa can be found in this zone in noticeable proportions. Habitats for deep-water chironomids, and oxygen concentrations in the lake, may also have been influenced by progressive infilling of the lake basin, which will have reduced the maximum depth in the lake center. However, the decrease of chironomid taxa that can colonize the lake profundal is largely attributed to the decrease of *Tanytarsus mendax*-type, a taxon that can also be very abundant in littoral conditions, and this decrease of profundal chironomid taxa must therefore be interpreted with caution.

In this section, the proportion of chironomid remains declines in favor of more Coleoptera and Ephemeroptera (Fig. 3). The increase of the latter order possibly indicates shallower waters (Luoto 2009). *Plumatella* rises in abundance. In small Swiss lakes in the Alpine region, high *Plumatella* values have been observed in relatively warm lakes with a tendency to hypoxic conditions where the abundances of deep-water invertebrate groups such as Chironomidae are reduced (Ursenbacher et al. 2020). Alternatively, high abundances of bryozoans such as *Plumatella* may also represent more habitats for this taxon, such as aquatic macrophytes in near-shore areas (Francis 2001). In agreement with this, Rhabdocoela oocytes, which are often considered as indicators of higher productivity and eutrophication (Haas 1996), show a noticeable increase in this zone. The lower axis 1 values of the CCA based on invertebrate remains (Fig. 4), indicative for lower bottom-water oxygen concentrations, also agree with less deep-water oxygen, which is also supported by the chironomid genera typical of eutrophic conditions such as *Chironomus*, *Ablabesmyia* and *Procladius* (Brodersen et al. 2004). Planktonic invertebrate remains are initially present at similar abundances as in earlier parts of the record, although they decrease towards the top of this zone (Fig. 4), a decrease which could also be explained by a shallowing of the lake due to infilling.

#### The late Holocene

In ZMN-4 (5150 cal yr BP-today) taxa with wide distributions such as *Tanytarsus mendax*-type and *Procladius* decrease (Fig. 2). This suggests changing environmental conditions favorizing better adapted specialists. Taxa indicative of relatively warm climate conditions, which were present in zones ZMN-2 and ZMN-3 (e.g. *Dicrotendipes*, *Einfeldia dissidens*/*natchitocheae*-type*, Cladotanytarsus mancus-*type, *Ablabesmyia, Tanytarsus pallidicornis*-type), are still present. *Chironomus plumosus*/*Einfeldia pagana*-type, *Paratanytarsus*, *Tanytarsus lactescens*-type and *Psectrocladius sordidellus-*type increase in abundance. These taxa are mainly indicators of eutrophic conditions except for some species of *Paratanytarsus* that typically live in oligotrophic waters (Brooks et al. 2007) and *Psectrocladius sordidellus*-type, which occurs over a wide nutrient gradient (Lotter et al. 1997, 1998). Other taxa appear for the first time and increase, such as *Polypedilum nubeculosum*/*nubifer*-type, *Glyptotendipes*, and *Heterotrissocladius marcidus*-type. *Polypedilum nubeculosum*/*nubifer*-type and *Glyptotendipes* are present in lakes at relatively high July air temperatures (Heiri et al. 2011) and eutrophic conditions (Brooks et al. 2007). Taxa that can colonize profundal habitats decrease even more whereas littoral taxa increase. This could again indicate a tendency to lower oxygen concentrations in the deepest part of the lake (Walker et al. 1993) or alternatively shallower in-lake conditions due to infilling of the lake basin. Interestingly, we also see a return of some taxa that were present in zones ZMN-1 and ZMN-2. *Tanytarsus lugens*-type, *Chironomus anthracinus*-type and *Microtendipes pedellus*-type increase again in the uppermost core section, possibly showing a recovery after a possibly human-induced eutrophication. Some of these taxa are also typical for cooler temperatures (Heiri et al. 2011). This change at the core top can also be observed in the increase in chironomid concentrations and in the proportion of the subfamily Orthocladiinae (Fig. 2).

The abundance of invertebrate remains varies little in ZMN-4 compared to ZMN-3 (Fig. 3). For example, Ephemeroptera are still present in high proportions and therefore indicate the presence of shallow waters (Luoto 2009). However, the proportion of invertebrates living in benthic habitats is highest in this zone (Fig. 3) suggesting that either the lake became shallower due to infilling, or littoral habitats increased relative to deep-water habitats. Rhabdocoela stay in high abundances possibly indicating high productivity and eutrophication as in zone ZMN-3 (Haas 1996). The relative abundance of *Plumatella* also increases, possibly indicating expanding habitats for this group, such as aquatic macrophytes. Axis 1 values of the CCA based on the aquatic invertebrate assemblages remain relatively low (Fig. 4), suggesting that the assemblages were typical for relatively low bottom oxygen concentrations in this zone (Ursenbacher et al. 2020). However, the values increase again in the topmost samples.

### Comparison with catchment vegetation and sedimentation rates

Chironomid-based temperature reconstructions rely on the assumption that chironomid assemblages are indicative for ambient temperature conditions and that the relationship between chironomid assemblages and temperature is not strongly distorted or biased by other drivers of ecosystem or community-composition change (Eggermont and Heiri 2012). Other mountain-lake records have shown that even early human activities such as pasturing can lead to substantial changes in chironomid assemblages, and thus, also of temperature reconstructions, therefore affecting their reliability (Heiri and Lotter 2005). Here we draw on the detailed reconstruction of past vegetation changes around Zminje Jezero that was developed in a parallel study based on pollen, plant macrofossils, and stomata from the same sediment record (Cagliero et al. 2023) to compare the temperature reconstruction to changes in vegetation and discuss possible influences of past human activities on Zminje Jezero based on past vegetation changes and sedimentation rates.

High pollen percentages (and presence of needles and stomata; Cagliero et al. 2023) indicate that *Pinus* dominated the vegetation both regionally as well as locally around the lake between 12,300 and 11,500 cal yr BP. The occurrence of a *Pinus*-dominated tree cover is typical of locations below the tree line during the Lateglacial (thus, including the YD) in southern Central as well as in Southeastern Europe (Rey et al. 2017; Lang et al. 2023). Colder and drier climate is confirmed by high percentages of Poaceae, *Artemisia* and total upland herb pollen typical for steppe/tundra (Fig. 4). This is in agreement with our chironomid-inferred temperature reconstruction showing colder temperature until 11,500 cal yr BP.

Vegetation composition shifted around 11,500 cal yr BP, both locally (expansion of *Betula*) as well as regionally (expansion of deciduous and rather mesophilous trees: *Quercus*, *Ulmus*, and *Fraxinus*). The latter is typical of early Holocene pollen records from the region (Lang et al. 2023) and agrees with the early Holocene rise of the chironomid-inferred temperature values. Interestingly, sedimentation rates dropped around 10,000 cal yr BP (Fig. 4) in conjunction with the start of the expansions of *Picea abies* and *Abies alba* in the surroundings, suggesting that increased forest cover contributed to reduce slope instability in the catchment. Forest cover remained high thereafter, first with a *Picea abies* and *Abies alba* dominated forest until 6400 cal yr BP and later with a mixed *Picea-Abies-Fagus* dominated forest until present day (Cagliero et al. 2023). In keeping with the persistent local occurrence of a dense forest cover, sedimentation rates in the lake were mostly low during the past 10,000 years. The rare signs of human activities documented by a single *Secale cereale* pollen finding starting from 5800 cal yr BP suggest that humans had very little influence on ecosystem dynamics locally (Fig. 4).

However, human activity became more noticeable from around 2000 cal yr BP onwards. *Olea europea*, *Juglans regia*, and *Castanea sativa* pollen abundances increased (Fig. 4) as these trees were cultivated at lower elevation along the Adriatic Sea coast. Moreover, cultural indicators (including *Plantago lanceolata*-type and Cerealia-type pollen) and secondary anthropogenic indicators (including *Plantago major*/*media*-type, *Urtica*-type, and *Rumex acetosa*) also increased starting from 2000 cal yr BP (Fig. 4). These insights in conjunction with first evidence of increasing sedimentation rates from ca. 3000 cal yr BP onwards, together with a more pronounced increase in sedimentation rates at ca. 2000 cal yr BP (Fig. 4), suggest that human activities may have influenced the lake ecosystem via increased catchment erosion. Indeed, late Holocene increases in sedimentation rates have been observed for other European mountain lakes where pasturing occurred in the catchment since the Bronze age (Thöle et al. 2016; Perret-Gentil et al. 2024). However, in the Zminje Jezero vegetation record there is no indication of major local human activities such as clearcutting or animal husbandry. Plant macrofossils and stomata of trees (*Picea, Abies,* and *Fagus*), which indicate the local presence of taxa (Ammann et al. 2014), were persistently found since at least 9500 cal yr BP, and dung fungi spores (*Sporormiella*) that are indicative for the presence of herbivores (Davis 1987) are absent for the Holocene. Conversely, the decrease of tree pollen centered on 500 cal yr BP and a substantial increase of pollen indicative of human activities from 500 cal yr BP to present may attest to the first settlements in the Durmitor Massif during the Middle Ages and associated land-use activities such as exploitation of timber and pasturing (Cagliero et al. 2023). For our record this implies that variations in the youngest interval of the chironomid-based temperature reconstruction should be interpreted with caution, as the increase in sedimentation rates from ca. 3000 cal yr BP and particularly the changes in vegetation from ca. 500 cal yr BP onwards may have been associated with significant influences of human activities on the lake and its environments.

### Interpretation of the chironomid-based temperature reconstruction

There is no local chironomid-temperature calibration dataset available for the Western Balkans. Nevertheless, the application of the Swiss calibration dataset and chironomid-temperature transfer function to Zminje Jezero resulted in a chironomid-based July air temperature record that features the main characteristics expected for the temperature development in this region, including relatively cool temperatures during the YD, higher mid- than late Holocene temperatures and a decrease in reconstructed temperature towards the latest Holocene. Compared with other chironomid-based Holocene temperature reconstructions from the Swiss Alps, the Apennines and the southern Carpathians (Heiri et al. 2015; Tóth et al. 2015; Samartin et al. 2017; Fig. 5), the main difference of the Zminje Jezero record is the unexpectedly warm early Holocene, with higher temperatures than recorded in the mid-Holocene, the relatively high temperatures that persist well into the late Holocene after a first temperature decrease around 11,000 cal yr BP, and the relatively late and stepwise decrease in latest Holocene temperatures at ca. 2000 cal yr BP. However, other chironomid-based reconstructions from the eastern Alps (Ilyashuk et al. 2011) and the northern Carpathians (Hájková et al. 2016; Fig. 5) also feature higher early Holocene temperatures than observed during the mid-Holocene, and the Zminje Jezero record therefore falls within the variation of early to mid-Holocene temperature development observed in other southern Central, Southern and Eastern European records. Similarly, the exact timing and amplitude of the mid-to late Holocene temperature decrease observed between chironomid records differs to some extent, with some records showing a gradual temperature decrease towards the late Holocene (Heiri et al. 2003) whereas others feature more stepwise changes (Heiri et al. 2015) or a combination of gradual and stepwise temperature decreases (Tóth et al. 2015). These differences may be associated with differences in temperature development across Europe, but also local hydrological and topographical effects and the sensitivity with which different lake ecosystems respond to gradual changes in temperature, which may also result in stepwise changes once certain ecosystem thresholds are passed.

The closest chironomid-based temperature reconstructions from the Mediterranean climate region are from two lakes in the northern Apennines (Italy), Gemini and Verdarolo (Samartin et al. 2017), situated close to each other at similar elevation (1349 and 1390 m a.s.l.) as Zminje Jezero (1535 m a.s.l). Chironomid-based temperature reconstructions from both of these lakes show a mid-Holocene thermal maximum between 9000 and 5000 cal yr BP with temperatures 1–2 °C higher than during the past 2000 years (Fig. 5). This is in accordance with our record also showing 0.3–1.5 °C higher temperatures in this same time period compared to the pre-industrial temperature levels. The timing of the beginning of the temperature decline after 5000 cal yr BP in these records and in our record also matches well. In contrast, both of these records show increasing temperatures during the earliest Holocene but not maximum values, although this may represent differences in the geographical and climatological setting between these lakes and Zminje Jezero.

Few other fully quantitative temperature reconstructions are available from the Western Balkans region though none based on fossil invertebrate remains. The closest site with a temperature reconstruction of the entire Holocene is ancient Lake Maliq (Albania), around 300 km from Zminje Jezero, a lake that has dried up in the past century because it was drained for agriculture. The pollen-based temperature reconstruction for this site (Bordon et al. 2009; Fig. 5) also features a pronounced increase in the mean temperature of the warmest month (MTWA) at the YD to Holocene transition. However, this increase is much more pronounced (ca. 8 °C, Bordon et al. 2009) than in any other reconstruction from the region, including the one from Zminje Jezero (Fig. 5). The increase in chironomid-inferred temperatures at Zminje Jezero agrees better with the estimated temperature change at this transition from other chironomid records from southern Central and Southern Europe (ca. 3–5 °C; Ilyashuk et al. 2011; Tóth et al. 2015; Hájková et al. 2016; Samartin et al. 2017). Further, the pollen-based temperature record from Lake Maliq only shows a relatively low variability in summer temperatures and lower temperatures in the mid-Holocene compared to the late Holocene, a feature that disagrees with Holocene summer insolation changes and has also been described for some other pollen-based temperature reconstructions from southern Europe (Samartin et al. 2017).

A further reconstruction of annual mean air temperature (MAT), based on methylation and cyclisation of branched tetraethers (MBT-CBT) indices is described by Thienemann et al. (2017) for Lake Dojran (North Macedonia/Greece), situated less than 400 km away from Zminje Jezero. Here the lowest temperature is observed at 11,500 cal yr BP during the YD which is in accordance with our findings. After this period, Thienemann et al. (2017) describe an increase of 3 °C during the early Holocene with a temperature maximum at 9500 cal yr BP. This maximum takes place somewhat later than in our reconstruction, where the relative thermal maximum, with reconstructed temperatures roughly 5 °C higher compared to the YD, takes place at 10,800 cal yr BP. However, overall the relative annual MAT variations for Lake Dojran follow a similar pattern as in our reconstruction during the Holocene (Thienemann et al. 2017; Fig. 5). Specifically, between 7500 and 5000 cal yr BP stable warm conditions are reconstructed as at Zminje Jezero. During the mid-Holocene/late Holocene transition, Thienemann et al. (2017) describe colder conditions that coincide with the dry and cold 4.2 kyr event. This event is apparently evident across the Mediterranean region and was followed at Lake Dojran by environmental instability at the start of the late Holocene as shown through distinct changes in some studied proxies (Francke et al. 2013). The temporal resolution of our record does not allow us to document such short-lived, abrupt temperature variations. However, the reconstructed temperatures from Zminje Jezero nevertheless follow a similar pattern as the annual MAT record from Lake Dojran during the last 1200 years, notably including a temperature drop of ca. 1 °C at 1000 cal yr BP followed by a minor temperature increase thereafter.

## Conclusions

The Zminje Jezero chironomid record provides evidence for a pronounced shift of chironomid-assemblage composition at the YD to Holocene transition, and more gradual changes within the Holocene. Chironomid and other aquatic invertebrate assemblages suggest meso- to eutrophic conditions over the entire analysed interval, with possibly slightly more oligotrophic conditions during the YD. The comparison of the invertebrate assemblages with those presently observed in small Swiss lakes (CCA axis 1 scores in Fig. 3) indicates that they remained typical for lakes with relatively high oxygen availability over the entire record. However, the assemblage composition from ca. 8400 cal yr BP until today (ZMN-3 and ZMN-4) is more characteristic of lakes with slightly reduced hypolimnetic oxygen conditions, suggesting, together with the occurrence of some chironomid taxa able to tolerate low oxygen conditions, that the lake may have experienced occasional phases of hypoxia particularly in the past ca. 5200 years (ZMN-4). Variations in the abundance of Ephemeroptera, littoral chironomid taxa and benthic invertebrate remains, particularly towards the late Holocene (ZMN-4), suggest that the lake may have experienced decreases in water depth possibly due to infilling of the basin. However, chironomid assemblages clearly remain indicative for aquatic conditions during the entire record, suggesting that these water-depth changes were limited. Applying a chironomid-temperature transfer function from Central Europe to the record resulted in a July air temperature reconstruction which agrees, in general features, with other chironomid-based temperature reconstructions from southern Central, Eastern and Southern Europe. It is characterized by relatively low reconstructed temperatures during the YD, relatively high temperatures in the early and mid-Holocene, with a distinct maximum in the earliest Holocene, and slightly lower values in the late Holocene. It shows a close agreement with another quantitative temperature reconstruction from Greece/North Macedonia (Lake Dojran) based on the MBT-CBT proxy, but also some differences with a pollen-based temperature reconstruction from the Balkan region (Lake Maliq), particularly regarding temperature stability during the Holocene. By showcasing the effectiveness of chironomid-based temperature reconstructions at Lake Zminje Jezero, we hope that this study will pave the way for future chironomid-based paleoclimate research in the Balkans.

## Supplementary Information

Below is the link to the electronic supplementary material.

Supplementary file1 (DOCX 136 KB)

Supplementary file2 (XLSX 12 KB)

Supplementary file3 (XLSX 22 KB)

Supplementary file4 (XLSX 16 KB)

## Data Availability

Novel data associated with this submission are included in the supplementary information files. Datasets published in Cagliero et al. 2023 (chronology, pollen, spores, stomata, plant macrofossils, charcoal and XRF records) are publicly available through the European Pollen Database (EPD) and can be accessed at the following 10.21233/9BC8-ZK56.

## References

[CR1] Ammann B, van der Knaap WO, Lang G, Gaillard M-J, Kaltenieder P, Rösch M, Finsinger W, Wright HE, Tinner W (2014) The potential of stomata analysis in conifers to estimate presence of conifer trees: examples from the Alps. Veget Hist Archaeobot 23:249–264

[CR2] Andersen T, Cranston PS, Epler JH (2013) Chironomidae of the Holarctic region: keys and diagnoses: larvae. Insect Syst Evol Suppl 66:1–571

[CR3] Bennett KD (1996) Determination of the number of zones in a biostratigraphical sequence. New Phytol 132:155–170 33863055 10.1111/j.1469-8137.1996.tb04521.x

[CR4] Blaauw M, Christen JA (2011) Flexible paleoclimate age-depth models using an autoregressive Gamma process. Bayesian Anal 6:457–474

[CR5] Bolland A, Kern OA, Allstädt FJ, Peteet D, Koutsodendris A, Pross J, Heiri O (2021) Summer temperatures during the last glaciation (MIS 5c to MIS 3) inferred from a 50,000-year chironomid record from Füramoos, southern Germany. Quat Sci Rev 264:107008

[CR120] Bordon A, Peyron O, Lézine A-M, Brewer S, Fouache E (2009) Pollen-inferred Late-Glacial and Holocene climate in southern Balkans (Lake Maliq). Quat Int 200:19–30

[CR6] Brodersen KP, Pedersen O, Lindegaard C, Hamburger K (2004) Chironomids (Diptera) and oxy-regulatory capacity: An experimental approach to paleolimnological interpretation. Limnol Oceanogr 49:1549–1559

[CR7] Brooks SJ (2003) Chironomid analysis to interpret and quantify Holocene climate change. In: Mackay A, Battarbee R, Birks J, Oldfield F (eds) Global change in the Holocene. Arnold, London, pp 328–341

[CR8] Brooks SJ, Langdon PG, Heiri O (2007) The identification and use of Palaearctic Chironomidae larvae in palaeoecology. QRA Technical Guide No. 10. Quaternary Research Association, London

[CR9] Burić D, Ducić V, Mihajlović J (2014) The climate of Montenegro: modificators and types—part two. Bull Serbian Geogr Soc 94:73–90

[CR10] Cagliero E, Morresi D, Paradis L, Curović M, Spalević V, Marchi N, Meloni F, Bentaleb I, Motta R, Garbarino M, Lingua E, Finsinger W (2022) Legacies of past human activities on one of the largest old-growth forests in south-east European mountains. Veg Hist Archaeobot 31:415–430

[CR11] Cagliero E, Paradis L, Marchi N, Lisztes-Szabó Z, Braun M, Hubay K, Sabatier P, Čurović M, Spalevic V, Motta R, Lingua E, Finsinger W (2023) The role of fire disturbances, human activities and climate change for long-term forest dynamics in upper-montane forests of the central Dinaric Alps. Holocene 33:827–841

[CR12] Campbell D, Humphries P, McCasker N, Nielsen D (2017) Subfossil chironomid head capsules reveal assemblage differences in permanent and temporary wetlands of southeastern Australia. Hydrobiologia 809:91–110

[CR13] Courtney-Mustaphi C, Steiner E, von Fumetti S, Heiri O (2024) Aquatic invertebrate mandibles and sclerotized remains in Quaternary lake sediments. J Paleolimnol 71:45–83

[CR14] Davis OK (1987) Spores of the dung fungus *Sporormiella*: Increased abundance in historic sediments and before Pleistocene megafaunal extinction. Quat Res 28:290–294

[CR15] Djurović P (2012) The Debeli Namet glacier from the second half of the 20th century to the present. Acta Geograph Slov 52:277–301

[CR16] Eggermont H, Heiri O (2012) The chironomid-temperature relationship: expression in nature and palaeoenvironmental implications. Biol Rev 87:430–456 22032243 10.1111/j.1469-185X.2011.00206.x

[CR17] Finsinger W, Morales-Molino C, Gałka M, Valsecchi V, Bojovic S, Tinner W (2017) Holocene vegetation and fire dynamics at Crveni Potok, a small mire in the Dinaric Alps (Tara National Park, Serbia). Quater Sci Rev 167:63–77

[CR18] Francis DR (2001) Bryozoan statoblasts. In: Smol JP, Birks HJB, Last WM (eds) Tracking Environmental Change Using Lake Sediments. Zoological Indicator, vol 4. Kluwer Academic Publishers, Dordrecht, pp 105–123

[CR19] Francke A, Wagner B, Leng MJ, Rethemeyer J (2013) A late glacial to Holocene record of environmental change from Lake Dojran (Macedonia, Greece). Clim past 9:481–498

[CR20] Gachev E, Mitkov I (2019) Small glaciers in Pirin (Bulgaria) and Durmitor (Montenegro) as glaciokarstic features. Similarities and differences in their recent behaviour. Quat Int 504:153–170

[CR121] Gelorini V, Verbeken A, van Geel B, Cocquyt C, Verschuren D (2011) Modern non-pollen palynomorphs from East African lake sediments. Rev Palaeobot Palynol 164:143–173

[CR21] Giesecke T, Davis B, Brewer S, Finsinger W, Wolters S, Blaauw M, de Beaulieu JL, Binney H, Fyfe RM, Gaillard MJ, Gil-Romera G, van der Knaap WO, Kunes P, Kuhl N, van Leeuwen JFN, Leydet M, Lotter AF, Ortu E, Semmler M, Bradshaw RHW (2014) Towards mapping the late Quaternary vegetation change of Europe. Veg Hist Archaeobot 23:75–86

[CR23] Gregory-Eaves I, Smol JP (2024) Chapter 30 - Paleolimnology: approaches and applications. In: Jones ID, Smol JP (eds) Wetzel’s Limnology, 4th edn. Academic Press, San Diego, pp 1015–1043

[CR24] Grimm EC (1987) CONISS: A Fortran 77 program for stratigraphically constrained cluster analysis by the method of incremental sum of squares. Comput Geosci 13:13–35

[CR122] Haas JN (1994) First identification key for charophyte oospores from central Europe. Eur. J. Phycol. 29:227-235

[CR25] Haas JN (1996) Neorhabdocoela oocytes- palaeoecological indicators found in pollen preparations from Holocene freshwater lake sediments. Rev Palaeobot Palynol 91:371–382

[CR26] Hájková P, Pařil P, Petr L, Chattová B, Grygar TM, Heiri O (2016) A first chironomid-based summer temperature reconstruction (13–5 ka BP) around 49°N in inland Europe compared with local lake development. Quat Sci Rev 141:94–111

[CR27] Heiri O, Lotter AF (2001) Effect of low count sums on quantitative environmental reconstructions: an example using subfossil chironomids. J Paleolimnol 26:343–350

[CR28] Heiri O, Lotter AF (2005) Holocene and Lateglacial summer temperature reconstruction in the Swiss Alps based on fossil assemblages of aquatic organisms: a review. Boreas 34:506–516

[CR29] Heiri O, Lotter AF (2010) How does taxonomic resolution affect chironomid-based temperature reconstruction? J Paleolimnol 44:589–601

[CR30] Heiri O, Millet L (2005) Reconstruction of late glacial summer temperatures from chironomid assemblages in Lac Lautrey (Jura, France). J Quat Sci 20:33–44

[CR31] Heiri O, Lotter AF, Hausmann S, Kienast F (2003) A chironomid-based Holocene summer air temperature reconstruction from the Swiss Alps. Holocene 13:477–484

[CR32] Heiri O, Brooks SJ, Birks HJB, Lotter AF (2011) A 274-lake calibration dataset and inference model for chironomid-based summer air temperature reconstruction in Europe. Quat Sci Rev 30:3445–3456

[CR33] Heiri O, Ilyashuk B, Millet L, Samartin S, Lotter AF (2015) Stacking of discontinuous regional palaeoclimate records: chironomid-based summer temperatures from the Alpine region. Holocene 25:137–149

[CR34] Heiri O, Brooks SJ, Birks HJB, Lotter AF (2019) NOAA/WDS Paleoclimatology—Swiss-Norwegian Chironomid-Temperature Calibration Dataset. NOAA National Centers for Environmental Information. 10.25921/bznn-sk22. Accessed on 16 Mar 2022

[CR35] Ilyashuk EA, Koinig KA, Heiri O, Ilyashuk BP, Psenner R (2011) Holocene temperature variations at a high-altitude site in the Eastern Alps: a chironomid record from Schwarzsee ob Sölden, Austria. Quat Sci Rev 30:176–191 21317974 10.1016/j.quascirev.2010.10.008 PMC3021123

[CR36] Janecek B, Moog O, Orendt C (2017) Diptera: Chironomidae: Podonominae & Buchonomyiinae. In: Moog O, Hartmann A (eds) Fauna Aquatica Austriaca 3. BMLFUW, Wien

[CR37] Jimenez-Moreno G, Heiri O, García-Alix A, Scott Anderson R, Jimenez-Espejo FJ, Lopez-Blanco C, Jimenez L, Perez-Martínez C, Rodrigo-Gamiz M, Lopez-Aviles A, Camuera J (2023) Holocene summer temperature reconstruction based on a chironomid record from Sierra Nevada, southern Spain. Quat Sci Rev 319:108343

[CR38] Juggins S (2007) C2: Software for Ecological and Palaeoecological Data Analysis and Visualisation (User Guide Version 1.5). Newcastle University, Newcastle upon Tyne

[CR39] Juggins S (2015) Rioja: analysis of Quaternary science data. https://CRAN.R-project.org/package=rioja

[CR40] Lang G, Ammann B, Behre K-E, Tinner W (eds) (2023) Quaternary vegetation dynamics of Europe. Haupt Verlag, Bern

[CR41] Lehmdal G (2000) Lateglacial and Early Holocene insect assemblages from sites at different altitudes in the Swiss Alps—implications on climate and environment. Palaeogeogr Palaeoclimatol Palaeoecol 159:293–312

[CR42] Lencioni V, Rossaro B (2005) Microdistribution of chironomids (Diptera: Chironomidae) in alpine streams: an autoecological perspective. Hydrobiologia 533:61–76

[CR43] Lotter AF, Birks HJB, Hofmann W, Marchetto A (1997) Modern diatom, cladocera, chironomid, and chrysophyte cyst assemblages as quantitative indicators for the reconstruction of past environmental conditions in the Alps. I. Climate J Paleolimnol 18:395–420

[CR44] Lotter AF, Birks HJB, Hofmann W, Marchetto A (1998) Modern diatom, cladocera, chironomid, and chrysophyte cyst assemblages as quantitative indicators for the reconstruction of past environmental conditions in the Alps. II. Nutrients. J Paleolimnol 19:443–463

[CR45] Luoto T (2009) An assessment of lentic ceratopogonids, ephemeropterans, trichopterans and oribatid mites as indicators of past environmental change in Finland. Ann Zool Fenn 46:259–270

[CR47] Mitchell EA, Charman DJ, Warner BG (2008) Testate amoebae analysis in ecological and paleoecological studies of wetlands: past, present and future. Biodivers Conserv 17:2115–2137

[CR48] Moller Pillot HKM (2009) Chironomidae Larvae Biology and ecology of the Chironomini, vol 2. KNNV Publishing, Zeist

[CR49] Moller Pilot HKM (2013) Chironomidae Larvae Biology and Ecology of the aquatic Orthocladiinae, vol 3. KNNV Publishing, Zeist

[CR50] Oksanen J, Blanchet FG, Friendly M, Kindt R, Legendre P, McGlinn D, Minchin PR, O’Hara RB, Simpson GL, Solymos P, Stevens MHH, Szoecs E, Wagner H (2020) Vegan: Community Ecology Package. R package version 2.5–7. https://cran.r-project.org/package=vegan. Accessed 23 Dec 2022

[CR51] Perret-Gentil N, Rey F, Gobet E, Tinner W, Heiri O (2024) Human impact leads to unexpected oligotrophication and deepwater oxygen increase in a Swiss mountain lake. Holocene 34:189–201

[CR52] Piasecki S (2022) Odd palynomorphs (NPPs) from annuli of fern sporangia; Holocene lacustrine and tsunami deposits of the Danish Wadden Sea. Palynology 46:1–8

[CR54] R Core Team (2021) R: A language and environment for statistical computing. R Foundation for Statistical Computing, Vienna, Austria. URL https://www.R-project.org/.

[CR55] Reimer PJ, Austin W, Bard E et al (2020) The IntCal20 Northern Hemisphere radiocarbon age calibration curve (0–55 cal kBP). Radiocarbon 62:725–757

[CR56] Rey F, Gobet E, van Leeuwen JFN, Gilli A, van Raden UJ, Hafner A, Wey O, Rhiner J, Schmocker D, Zünd J, Tinner W (2017) Vegetational and agricultural dynamics at Burgäschisee (Swiss Plateau) recorded for 18,700 years by multi-proxy evidence from partly varved sediments. Veg Hist Archaeobot 26:571–586

[CR57] Rieradevall M, Brooks SJ (2001) An identification guide to subfossil Tanypodinae larvae (Insecta: Diptera: Chironomidae) based on cephalic setation. J Paleolimnol 25:81–99

[CR58] Saether OA (1979) Chironomid communities as water quality indicators. Holarct Ecol 2:65–74

[CR59] Samartin S, Heiri O, Joos F, Renssen H, Franke J, Brönnimann S, Tinner W (2017) Warm Mediterranean mid-Holocene summers inferred from fossil midge assemblages. Nat Geosci 10:207–212

[CR60] Schmid PE (1993) A key to the larval Chironomidae and their instars from Austrian Danube region streams and rivers with particular reference to a numerical taxonomic approach. Part I. Diamesinae, prodiamesinae and orthocladiinae. Wasser und Abwasser Suppl 3(93):1–514

[CR62] Solhøy T (2001) Orbatid mites. In: Smol JP, Birks HJB, Last WM (eds) Tracking Environmental Change Using Lake Sediments. Zoological Indicators, vol 4. Kluwer Academic Publishers, Dordrecht, pp 81–104

[CR64] Taylor KJ, Potito AP, Beilman DW, Ghilard B, O’Connell M (2013) Palaeolimnological impacts of early prehistoric farming at Lough Dargan, County Sligo, Ireland. J Archaeol Sci 40:3212–3221

[CR65] Ter Braak CJ, Juggins S (1993) Weighted averaging partial least squares regression (WA-PLS): an improved method for reconstructing environmental variables from species assemblages. Twelfth International Diatom Symposium. Springer, Dordrecht, pp 485–502

[CR66] Thienemann M, Masi A, Kusch S, Sadori L, John S, Francke A, Wagner B, Rethemeyer J (2017) Organic geochemical and palynological evidence for Holocene natural and anthropogenic environmental change at Lake Dojran (Macedonia/Greece). Holocene 27:1103–1114

[CR67] Thöle L, Schwörer C, Colombaroli D, Gobet E, Kaltenrieder P, van Leeuwen J, Tinner W (2016) Reconstruction of Holocene vegetation dynamics at Lac de Bretaye, a high-mountain lake in the Swiss Alps. Holocene 26:380–396

[CR68] Thorp JH, Rogers DC (2014) Ecology and general biology thorp and Covich’s freshwater invertebrates, vol 1. Elsevier, Amsterdam

[CR69] Tinner W, Conedera M, Ammann B, Gaeggler HW, Gedye S, Jones R, Saegesser B (1998) Pollen and charcoal in lake sediments compared with histotically documented forest fires in southern Switzerland since AD 1920. Holocene 8:31–42

[CR70] Tóth M, Magyari EK, Buczkó K, Braun M, Panagiotopoulos K, Heiri O (2015) Chironomid-inferred Holocene temperature changes in the South Carpathians (Romania). Holocene 25:569–582

[CR71] Ursenbacher S, Stötter T, Heiri O (2020) Chitinous aquatic invertebrate assemblages in Quaternary lake sediments as indicators of past deepwater oxygen concentration. Quat Sci Rev 231:106203

[CR72] Vallenduuk HJ, Moller Pillot HKM (2007) Chironomidae Larvae—General Ecology and Tanypodinae, vol 1. KNNV Publishing, Zeist

[CR73] van der Knaap WO, van Leeuwen JFN, Fankhauser A, Ammann B (2000) Palynostratigraphy of the last centuries in Switzerland based on 23 lake and mire deposits: chronostratigraphic pollen markers, regional patterns, and local histories. Rev Palaeobot Palynol 108:85–142 10.1016/s0034-6667(01)00049-5 11389919

[CR74] Vandekerkhove J, Declerck S, Vanhove M, Brendronck L, Jeppesen E, Conde Porcuna JM, De Meester L (2004) Use of ephippial morphology to assess richness of anomopods: potentials and pitfalls. J Limnol 63:75–84

[CR75] Walker IR (1987) Chironomidae (Diptera) in Paleoecology. Quat Sci Rev 6:29–40

[CR76] Walker IR (2001) 3. Midges: Chironomidae and related Diptera. In: Smol JP, Birks HJB, Last WM (eds) Tracking Environmental Change Using Lake Sediments, vol 4: Zoological Indicators. Kluwer Academic Publishers, Dordrecht, pp 43–66

[CR77] Walker IR, Reavie ED, Palmer S, Nordin RN (1993) A palaeoenvironmental assessment of human impact on Wood Lake, okanagan valley, British Columbia, Canada. Quat Int 20:51–70

