# Supplementary material for: Holocene ecosystem and temperature development inferred from invertebrate remains in Zminje Jezero (Dinaric Alps, Montenegro)
Source: J Paleolimnol. 2024 Aug 6;72(3):343–61. doi: 10.1007/s10933-024-00334-y (PMC11422249; doi:10.1007/s10933-024-00334-y)
Supplement: Supplementary file 1 — Supplementary file1 (DOCX 136 KB) [file 10933_2024_334_MOESM1_ESM.docx]

**Supplementary Information**

**
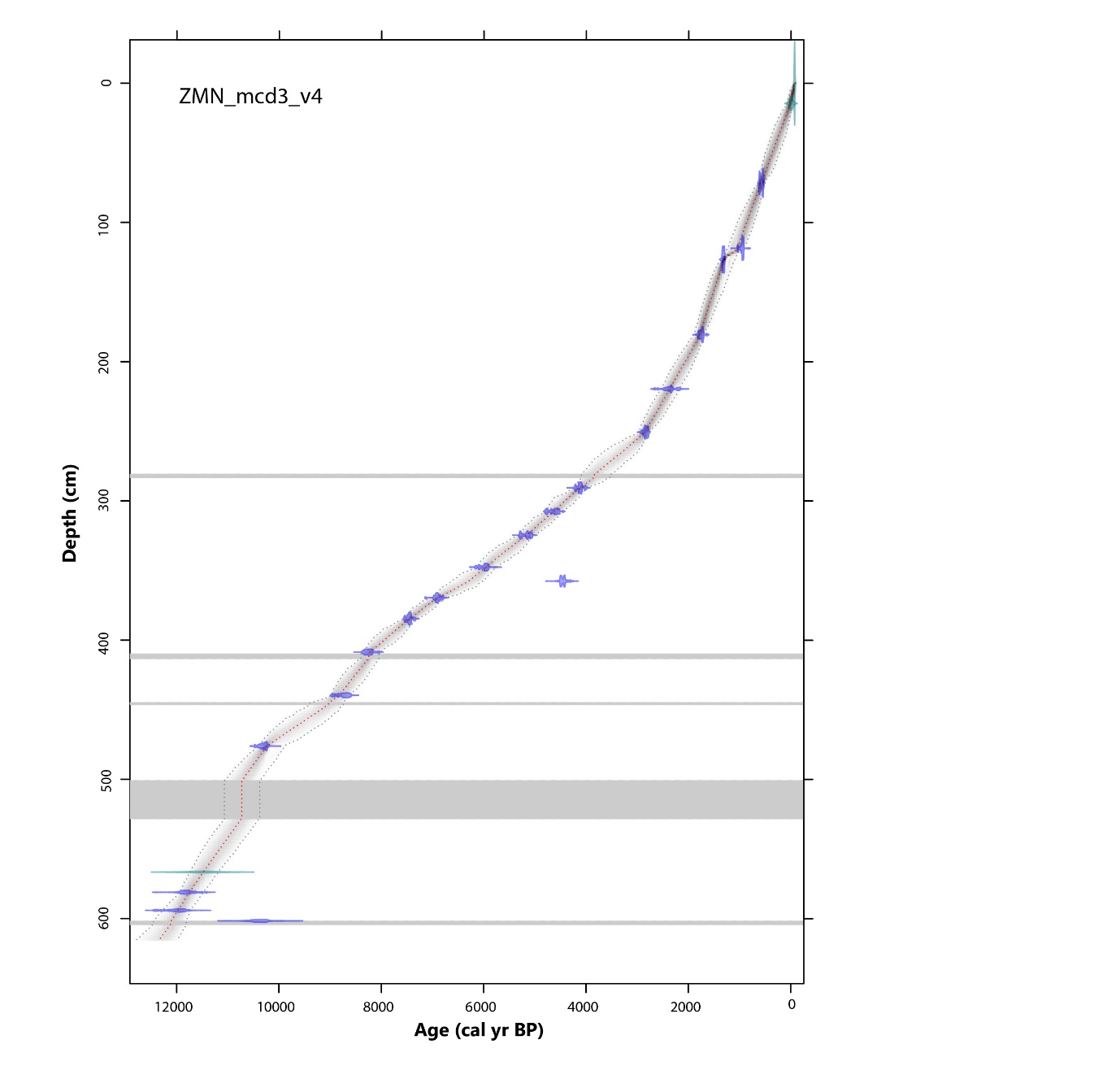
**

**Fig. S1** Age-depth model used in this study for the Zminje Jezero sediment core whose median sample ages slightly differ (maximum 60 cal yr BP) from the ages of the model used in Cagliero et al. (2023). Horizontal grey bands indicate instantaneous sedimentation events (turbidites) excluded from the composite section.

**Table S1** All dates including ^14^C dates and calibrated ages from the Zminje Jezero record used for the age-depth model for this study (from Cagliero et al. 2023).

| Lab. code/Name | Depth (cm) | Material | ^14^C age (yr BP) | ^14^C age error interval (yr) | Median age (cal yr BP)^a^ | Age 2σ range (cal yr BP)^a^ | Rejected from the age-depth model by RBacon |
| --- | --- | --- | --- | --- | --- | --- | --- |
| Surface | 0 |  | -69 | 5 | -69 |  |  |
| *Ambrosia*-type | 14.5 |  | 0 | 30 | 0 |  |  |
| DeA-33892^b^ | 7.5 | Terrestrial plant macrofossils | 565 | 19 | 599 | 531 - 627 |  |
| DeA-23961^b^ | 118.5 | Terrestrial plant macrofossils | 1054 | 24 | 948 | 921 - 1051 |  |
| DeA-32618^b^ | 126.5 | Terrestrial plant macrofossils | 1427 | 20 | 1327 | 1298 - 1349 |  |
| DeA-31441^b^ | 180.5 | Terrestrial plant macrofossils | 1844 | 23 | 1787 | 1724 - 1866 |  |
| DeA-33893^b^ | 219.5 | Terrestrial plant macrofossils | 2335 | 55 | 2359 | 2154 - 2687 |  |
| DeA-31442^b^ | 250.5 | Terrestrial plant macrofossils | 2755 | 22 | 2833 | 2778 - 2925 |  |
| DeA-31091^b^ | 290.5 | Terrestrial plant macrofossils | 3762 | 22 | 4123 | 3997 - 4233 |  |
| DeA-23962^b^ | 307.5 | Terrestrial plant macrofossils | 4108 | 30 | 4628 | 4454 - 4815 |  |
| DeA-31093^b^ | 324.5 | Terrestrial plant macrofossils | 4532 | 25 | 5156 | 5051 - 5312 |  |
| DeA-33890^b^ | 347.5 | Terrestrial plant macrofossils | 5212 | 51 | 5976 | 5896 - 6181 |  |
| DeA-23963^b^ | 357.5 | Terrestrial plant macrofossils | 3980 | 30 | 4468 | 4304 - 4525 | yes |
| DeA-31440^b^ | 369.5 | Terrestrial plant macrofossils | 6060 | 28 | 6913 | 6796 - 7145 |  |
| DeA-23964^b^ | 384.5 | Terrestrial plant macrofossils | 6552 | 37 | 7463 | 7362 - 7565 |  |
| DeA-33891^b^ | 408.5 | Terrestrial plant macrofossils | 7449 | 60 | 8267 | 8050 - 8385 |  |
| DeA-23967^b^ | 439.5 | Terrestrial plant macrofossils | 7908 | 39 | 8729 | 8597 - 8981 |  |
| DeA-23965^b^ | 476 | Terrestrial plant macrofossils | 9139 | 44 | 10,296 | 10,223 - 10,485 |  |
| HOL-begin | 566.5 |  | 11500 | 250 | 11,500 |  |  |
| Poz-10170^c^ | 581 | Terrestrial plant macrofossils | 10,170 | 60 | 11,814 | 11,404 - 12,039 |  |
| Poz-143126^c^ | 594 | Terrestrial plant macrofossils | 10,260 | 60 | 11,987 | 11,754 - 12,457 |  |
| Poz-143573^c^ | 601.5 | Terrestrial plant macrofossils | 9190 | 120 | 10,381 | 9968 - 10,702 | yes |

^a^ calibrated using the IntCal20 Northern Hemisphere radiocarbon age calibration curve (Reimer et al. 2020)

^b^ Laboratory Isotopotech Zrt. in Debrecen (Hungary)

^c^ Poznan Radiocarbon Laboratory (Poland)
